# Phasic spiking in vasopressin neurons: How and Why

**DOI:** 10.1111/jne.13042

**Published:** 2021-11-08

**Authors:** Duncan J. MacGregor

**Affiliations:** ^1^ Centre for Discovery Brain Sciences University of Edinburgh Edinburgh UK

**Keywords:** modelling, patterning, phasic firing, secretion, spiking, vasopressin

## Abstract

The plain title might have been an almost retro sounding grumpy retort, but it has inspired a journey of sorts, and something along the way I hope you won’t have come across before. An opinionated exploration of the distinctive phasic spiking patterns of magnocellular vasopressin neurons of the supraoptic and paraventricular nuclei of the hypothalamus. A mostly life essential population of neurons that signal the kidneys to regulate water loss in response to signals that encode plasma volume and osmotic pressure, as well as regulating blood pressure, and possibly metabolism and social behaviour. The viewpoint of a modeller shorn of any explicit maths.

## Graphical abstract

A description and discussion of the mechanism and purpose of the distinctive phasic patterned spiking of the magnocellular vasopressin neurons of the hypothalamus.

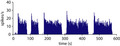

## 1 THE PSYCHOGEOGRAPHY OF THE PHASIC BURST (AND SILENCE)

One of the sad things about phasic spiking is that no one outside the individual neuron ever gets to see it, at least until the electrophysiologists come along and stick their electrodes in. This hidden internal nature of spiking is true of most neurons and presents an ongoing challenge for the invasive viewers to reconcile what they see and record with function; but electrical activity is so show‐off‐able and seductive, and phasic spiking is especially beautiful.

Silence. Occasional single spikes might pierce the darkness and flicker away, but really amounting to nothing other than indicating that something is still there. Just enough to add anticipation to a long anticipatory pause. Then a few coincide, perhaps just a tentative two or three, but rapidly followed, too many to distinguish now, and then suddenly a piercing rapid brightness transitions the darkness into pulsing random white noise. Occasional flickering hesitations, tripping from running too fast, picked up again, charging on. But then a longer stutter, slowing, single pulses distinguishable again and then a final spike unfollowed. In the slowness anticipation takes a moment to accept the silence, but then it is. Back in the darkness, with even a sense of relief.

**FIGURE 1:**
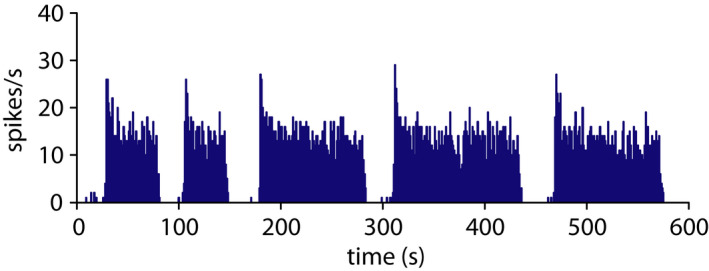
Phasic spiking in a vasopressin neuron. A typical example recorded *in vivo* using an extracellular electrode inserted into the supraoptic nucleus of the hypothalamus of a rat under urethane anaesthesia. The recorded spike times are plotted as counts of spikes s^‐1^ showing a distinctive pattern of bursts and silences with very sharp shifts between the two states

It is such a complex and elegant thing to exist in isolation and strange to describe just in words. Most commonly, we observe phasic spiking in pictures (Figure 1) but the best medium to get a sense from is sound (see Supporting information, S1), as on the electrophysiologists rig. The viewer that matters most is the voltage‐activated Ca^2+^ channels of the neuron's secretory axonal terminals, and this summed Ca^2+^ signal probably has more in common with our perception of sound than vision.

How and why in neuroscience and physiology are usually tied together, but not always in predictable ways, and early on in investigations it favours a tractable approach to choose one.

## 2 HOW DOES PHASIC SPIKING WORK?

Bursting is exceptionally common in the electrical activity of neurons, perhaps more common than not. In general, the mechanism that generates bursts uses positive feedback. If, when one spike is fired, the generation of another spike becomes more likely, then this will produce bursting. Equally important is some resistance, something must stop the burst or else it will just become continuous. This may be another mechanism that actively opposes spiking (such as an accumulating hyperpolarisation), or a weakening of the positive feedback mechanism. Phasic spiking is remarkable because the bursts are so stable. They last for tens of seconds and contain hundreds of spikes. Typical bursts are often just a few spikes or last a few seconds at most. It is also distinctive for its prolonged silences, usually shorter than the bursts, but still often lasting as long as 20 s. Hence the silences are also a stable state, and spike generation is considered *bistable*. Stability is always relative of course, but here it indicates that there are mechanisms acting on the timescale of tens of seconds to stabilise spiking activity (tens of milliseconds). To understand how phasic spiking works requires an understanding of how these states are made stable, and how they are switched between.

Very many of the mechanisms of neurons, and physiology in general, are concerned with bridging timescales. To be responsive to change requires signals that are transitory, but to maintain that response requires signals that are sustained, acting as a memory or summation of those transitory signals. This is the purpose of Ca^2+^ signalling in neurons. Ca^2+^ in neurons is highly complex, subject to mechanisms of entry, clearance and buffering that vary across the neuron, both spatially and temporally. There are thus many different Ca^2+^ signals, of varied magnitudes and timescales. One way to deal with this is to consider each of its functional roles as separate, communicating mechanisms.

Phasic bursts are Ca^2+^ dependent.^1^ Ca^2+^ signals modulate the electrical activity of neurons by controlling and sustaining hyperpolarising or depolarising voltages and currents. Depolarisation shifts a neuron's membrane potential closer to its spiking threshold and, if this depolarisation is spike activity‐dependent, then it can act as the positive feedback mechanism to generate bursting. Such a depolarisation that follows a spike is called a depolarising afterpotential (DAP) and these are commonly sensitive to voltage or Ca^2+^, or both. A DAP sufficiently long lasting to sustain phasic bursting would be expected to be Ca^2+^‐dependent. Thus forms an idea of how phasic spiking works; a slow Ca^2+^‐dependent DAP driven by the summed contributions of multiple spikes, to produce a self‐sustaining ‘plateau potential’.^2^, ^3^

The opposing mechanism uses another activity‐dependent signal, which acts on an even slower timescale than the Ca^2+^ signal; dendritically released dynorphin.^4^ This acts back on the secreting neuron (as an autocrine signal) to reduce the Ca^2+^ sensitivity of the phasic burst generating mechanism.^5^, ^6^ The different timescale is important because, if two signals have opposing actions and act on the same timescale, then they will mostly cancel out. Dynorphin takes longer to accumulate than the Ca^2+^ signal, but eventually will dominate, degrading the burst sustaining positive feedback and causing the burst to fail.

Modelling serves both the *how* and *why*. In serving *how*, it formalises and assembles the experimentally observed components, and tests whether these are sufficient to explain the experimentally observed behaviour. If they do not appear to be sufficient, then it can suggest the form of the missing parts. In serving *why*, it is used to simulate, and accessibly manipulate, the observed behaviour, providing a tool for testing its purpose. Ideally, these two will go hand in hand, but motivations and fortunes will often shift the balance between the two.

**FIGURE 2:**
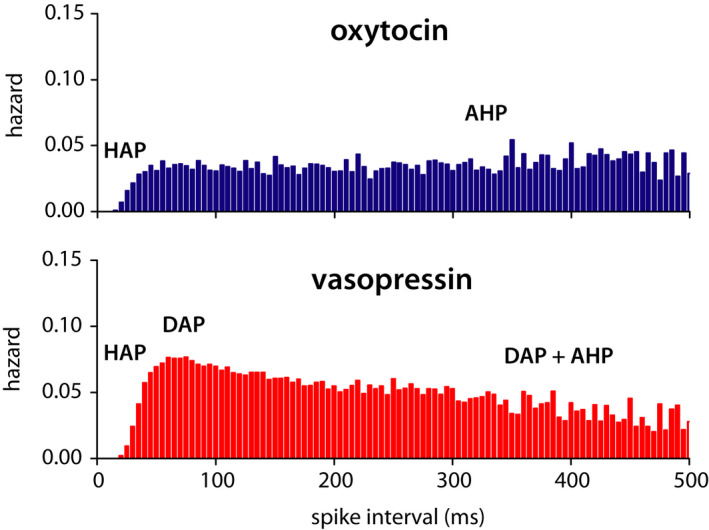
Typical hazard functions in oxytocin and vasopressin neurons. The hazard function plots the change in excitability following a spike firing measuring short‐timescale spike patterning, shaped mainly by a combination of spike‐triggered hyperpolarising and depolarising afterpotentials. AHP, afterhyperpolarisation; DAP, depolarising afterpotential; HAP, hyperpolarising afterpotential

Such is the interest, there have been many attempts to model phasic spiking. All of these are centred on assembling simplified (to varying degrees) representations of the set of activity‐dependent hyperpolarising and depolarising mechanisms, which shape the spike patterning of vasopressin neurons. A potent tool for measuring and matching this spike patterning is the analysis of inter‐spike intervals (ISIs), using both the *ISI histogram* and the *hazard function*. The hazard function processes the histogram to turn its ISI distribution into a plot of how the chance of spiking (excitability) changes with post‐spike time (Figure 2). The limitation is that ISI analysis only measures effects on the timescale of single spike intervals. These of course vary, but longer intervals are fewer, and its useful timescale is usually no more than 1 s.

The simplest feature observed here, which is common to many neurons, is the hyperpolarising afterpotential (HAP).^7^ This large magnitude but rapidly decaying hyperpolarisation generates a post‐spike refractory period, manifesting in the histogram as a lack of short intervals, and in the hazard as an initially zero chance of spiking, which recovers over the first few tens of milliseconds. The hazard of a simply patterned neuron will recover to a flat plateau where the chance of spiking becomes unaffected by time and is essentially random (top panel, Figure 2). Vasopressin neurons, however, have a distinctive peak of increased excitability that follows the refractory HAP, generated by a DAP. *The* DAP? Simple modelling can be used to match the ISI histogram and hazard, with the HAP and DAP defined by their magnitude per spike and decay rate. Typically (fitting the nice neat looking recordings favoured by early attempts at modelling), the decay rates are around 8 ms and 200 ms, respectively. Testing such a DAP in the model was indeed able to generate bursts, but not make them stable or sustained. The necessary random gaps, in synaptic input, and the resulting spiking, were sufficient as an opposing force. These are necessary because, even in a phasic burst, spike timing retains a large random component to its patterning. Making the DAP of sufficient magnitude to bridge these random gaps, as well as sustain a burst, also removes this randomness.

Incorporating dynorphin into the model has no purpose without a sustained burst for it to oppose, and so models added artificial bistability, using a purely abstract mathematical form. This works, reproducing the stable bursts and silences, as well as the transitions between these states, to fairly closely mimic recorded neurons.^8^ It breaks, however, when the extra dimension of changing input activity is added. The artificial mechanism is a good mimic, but it is brittle, and cannot flex with the changing neuron.

The essential realisation is that there are two DAPs. The DAP that appears in the limited timescale detection of the hazard is a fast DAP. It certainly contributes to bursting, but it is not *the* DAP that maintains the burst state. Its decay rate is simply too fast to resist the short timescale variations in input and spiking activity. There are two proposed mechanisms for a slow DAP: a depolarisation conducted by some Ca^2+^ sensitive non‐specific cation channel,^9^, ^10^ and not a depolarisation but the switching‐off of a hyperpolarisation generated by a K^+^ leak current.^11^ A positive generated by switching off a negative. Is there a functional difference?

The often neglected element is the silence. A simple model using conventional fast and slow DAPs, the latter modulated by dynorphin, can produce long bursts, but it cannot match the almost absolute silence of in vivo recorded vasopressin neurons. The conventional slow DAP, which is switched off by dynorphin, has no power to resist the continuing synaptic stimulation. It can be made to produce alternating slow and fast firing by combining a very hyperpolarised resting potential with a low rate of random synaptic input, occasionally able to summate sufficiently to activate the DAP plateau and produce a sustained burst, but it is not robust. The balance between parameters to successfully produce bursts is delicate, and the transitions between burst and silence do not resemble the dramatic switching of recorded phasic neurons. It also cannot match the short‐term spike patterning captured by the hazard function. When a sustained plateau depolarisation forms the major component of achieving spiking threshold, then the spike intervals tend to be much more regular than the mostly random patterning observed in the intra‐burst spiking of recorded neurons, similar to the difference in vitro, where a sustained depolarising stimulus is applied.

Both the problems of silence and spike patterning are solved by replacing the slow DAP with the switching off of a hyperpolarisation. The default hyperpolarised state produces almost silence, even when subject to substantial rates of depolarising synaptic input. When a random short burst of activity does accumulate sufficient Ca^2+^ to switch off the hyperpolarisation and shift into a burst, then the spiking, driven mostly by synaptic input instead of some plateau potential, retains the randomness that matches the recorded patterning. The ‘burst’ essentially, is actually normal spiking rather than a burst, comparable in its spike interval patterning to the continuous spiking observed in non‐phasic vasopressin and oxytocin neurons.

**FIGURE 3:**
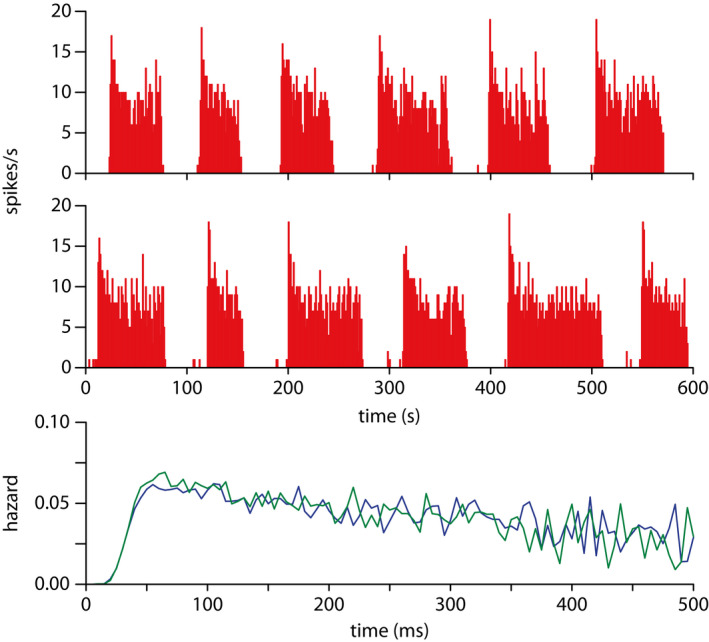
In vivo and model generated phasic spiking. Can you guess which is which? One spike rate panel shows integrate‐and‐fire based model generated data; the other shows the in vivo recorded fitted cell. Lower: overlaid model and cell generated hazard functions. The fitted model closely matches both the long and short timescale spike patterning

Although the K^+^ leak current functioning in this role is yet to be conclusively demonstrated experimentally, the modelling applied in different forms to data recorded in vitro ^12^ and in vivo ^13^ shows that it is the simplest and most robust idea for how phasic firing works. The simple in vivo model produces spike patterning that is indistinguishable from a recorded neuron (Figure 3), including both the dramatic head of the burst, the following increasingly unstable hinterland, and the anticipatory longing silences. The bistability uses no artificial component but is instead ‘emergent’, produced by the complex interaction of random synaptic input and competing positive and negative feedbacks. The bistability itself is activity‐dependent, supple and robust, and matches the changes in behaviour observed in recorded neurons subject to changing inputs. It thus serves both as explanation of how, and as a tool to examine *why*.

## 3 WHY PHASIC SPIKING?

The first essential principle when looking at the function of neurons, is that their output is not spiking, it is secretion. Excepting the few special cases of things such as gap junctions, which doesn't apply here. This would not be an important point except that the relationship between spiking and secretion is usually highly non‐linear (i.e., a single spike does not simply correspond to a single unit of secretion). Neuroendocrine neurons, and in particular vasopressin and oxytocin neurons, are an excellent model for studying secretion because their secretion mechanisms are much bigger and secrete larger, more measurable quantities compared to the most common secretion sites, at synapses. The two dominant non‐linear dynamics of the spike‐secretion coupling in vasopressin neurons are frequency facilitation, and fatigue.^14^ In frequency facilitation, the rate of secretion per spike increases with spike frequency. Thus more spikes, and more secretion per spike, produces a steep non‐linear increase in the rate of secretion. It also means that secretion is sensitive to patterning, as well as the rate of spiking. Regularly spaced spikes produce a smaller secretion response than a bursting, or simply noisier, pattern of the same mean spiking rate that more commonly produces shorter spike intervals.^15^

Fatigue acts over a longer timescale of tens of seconds, such that the rate of secretion drops in response to sustained stimulation. Fatigue is a common property of secretory mechanisms, which usually have a limited store of readily releasable peptide (or other transmitter) containing vesicles docked at the secretory membrane. These stores and the ability to secrete will be depleted if the stimulated rate of secretion exceeds the usually slower rate of replenishment. However, in vasopressin neurons, the fatigue dynamic appears to be ‘artificial’, in that the secretion response becomes fatigued before the releasable stores are depleted. This is a result of a down‐regulation of the Ca^2+^ entry response to spikes arriving at the secretory terminals.^16^ This artificial fatigue appears to act to protect the mechanism from the real fatigue of depleted stores.^17^ Indeed, looking more broadly at physiology, this appears to be a common theme; mechanisms of artificial fatigue (or pain sensing) that protect against lasting damage or depletion.

The optimal frequency range and timescale of fatigue in the stimulus‐secretion coupling of vasopressin neurons is such that phasic spiking produces an optimal secretion response per spike.^18^ The bursts maximise frequency facilitation, and the silences allow recovery from fatigue. This is the classic explanation of the purpose of phasic spiking, and it is a strong one, but it has problems. Is spiking optimised for the secretory mechanism or is the secretory mechanism optimised for the spiking? Why would optimising secretion per spike be important?

## 4 POPULATION SIGNALLING

The second essential principle is that functional signals are produced, not by single neurons, but by large populations or networks of neurons. Phasic patterning, even what remains after the filtering of the non‐linear secretion response, is never seen beyond the neuron because the vasopressin neurons are asynchronous^19^ and the functional signal is the summed secretory output of thousands of neurons. What is also important here is the dynamics of the pool that the signal is being secreted into. The combined processes of diffusion, between the plasma and extra vascular fluid, and clearance, mainly at the liver and kidneys,^20^ result in a half‐life of around one to two minutes, which further blurs any short timescale patterning in the neuron secreted signals. Thus, the bursting is not preserved in the functional vasopressin signal. This is different from several other endocrine signals such as growth hormone and corticotrophin‐releasing hormone where pulsatile patterning is essential to function, requiring synchronisation of the directing neurons. Oxytocin neurons have both modes of action, synchronising as a network to generate large pulses to trigger lactation or parturition, and acting as an asynchronous population to generate a slower changing modulatory signal, similar to vasopressin.

Using fewer spikes might be important. The vasopressin signal in its homeostatic role controlling water loss at the kidneys has to be maintained for life‐long periods of time and, as such, needs to be very robust and efficient. Neurons will die and are more likely to die when they are more active. Spiking also consumes energy, tiny amounts per neuron, but this might be significant in a population of thousands (around 10,000 in rats and 100,000 in humans) on this timescale.

But is something as elaborate as the generation of phasic spiking and its symbiosis with the secretory mechanisms required to reduce the number of spikes used? Oxytocin neurons are a useful comparison because they have very similar secretory mechanisms but different stimulus‐secretion coupling properties.^21^ The optimal frequency range goes much higher, and they show less fatigue. Thus these properties are not fixed by the nature of the secretory mechanism. The differences likely relate to the different functional roles. Vasopressin mainly requires endurance and oxytocin not only requires some endurance, but also the ability to sprint. Vasopressin does occasionally need to rapidly up‐regulate in its role maintaining blood pressure but not to the extreme of pulsatile oxytocin release.

As well as energy, the population of neurons depends on the extracellular environment, and the maintenance of ionic gradients. Asynchronous activity will avoid producing large amplitude global fluctuations in these, particularly K^+^, Ca^2+^ and Cl^−^.

## 5 SIGNAL PROCESSING

If phasic spiking does not shape the output signal, does it shape the processing of the input signal? The bistability of the phasic spiking mechanism conveys interesting signal processing properties. It can be demonstrated experimentally that a short intense stimulus can either initiate or terminate phasic bursts, depending on whether it falls during an ongoing burst or silence.^22^ Across the asynchronous population, this results in some neurons increasing and others decreasing their activity. One purpose of this might be to act as a low‐pass filter, reducing the response to transient changes in the input signal that might be noise. Modelling shows that, as well as reducing (but not removing) the response to transient signals, it also produces, as a population, a more consistent response to transient signals than non‐phasic neurons, independent of a slower changing background signal.^13^ Thus phasic spiking maintains the ability to independently respond to signals on two different timescales.

**FIGURE 4:**
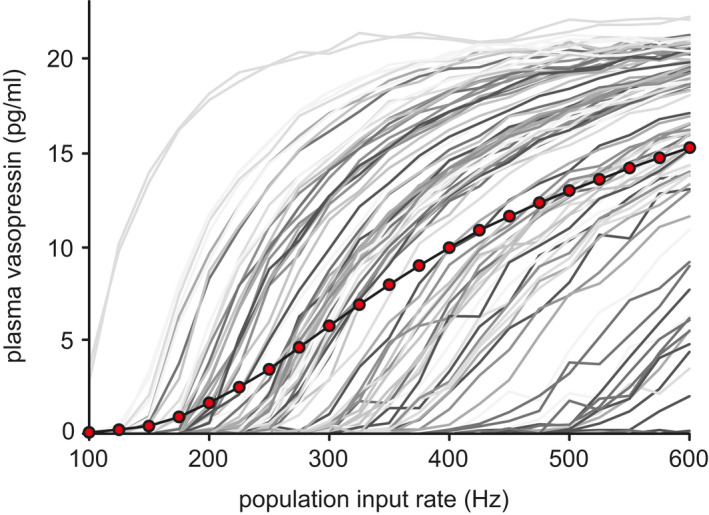
Heterogeneous population signal response. Stable plasma concentrations produced by a population of 100 coupled spiking and secretion model neurons with heterogeneous synaptic input input rates. The grey traces show the scale normalised secretion responses of the individual neurons in the population. The red dots show the summed population signal. The randomly varied individual non‐linear responses sum to produce a much more linear response signal

A related property is how the activity of a phasic neuron changes in response to a slow‐changing input signal. Neurons by default produce a very non‐linear (in simplest terms, switch‐like) response to an increasing input signal, based on the action potential, which is an all or nothing event. The spiking rate of a non‐phasic neuron will tend to bend up in a curve in response to a linear increase in input.^23^ Phasic neurons, however, produce a much more linear response, and this is particularly useful in a homeostatic role, where the ideal signal response is linear. To understand this, imagine being in control of a heater maintaining the temperature of a vessel of water. If your response is very non‐linear (i.e., you can only switch the heater on and off), then it is very difficult to maintain, resulting in a temperature that oscillates up and down. A smooth, consistently proportional, response to changes in temperature will give much better control. There are several other electrophysiological properties of vasopressin neurons that also contribute to a more linear spiking response, including the mixed excitatory and inhibitory synaptic coding of the input signal, as well as the afterhyperpolarisation (AHP).^24^ However, the linear spiking response is disrupted by the non‐linear coupling to secretion. What does restore the linear plasma signal response of the population is heterogeneity of activity across the neurons.^17^ The many different non‐linear secretion responses sum together to form a robust linear population response (Figure 4). A similar effect can be demonstrated in modelling non‐phasic oxytocin neurons. Thus, although phasic spiking contributes to generating a linear signal response, it is not essential. The dominant factor is the heterogeneity.

## 6 CONCLUSIONS

We don't have a satisfactory *why* yet, but it appears likely that something so elegant and requiring such effort has purpose. Finding this will require going beyond the seductive recordings to looking at the functional signal processing of these neurons acting as a coordinated population, as part of a physiological system. The apparently minor effect of the medium AHP in reducing spike rate variability in oxytocin neurons, for example, appears to be much more substantial when considering its effect on stabilising secretion and information coding as a population.^15^ More answers might lie in the *how* and *why* of dendritic communication between the vasopressin neurons. We know many parts of the mechanisms here, but not much of the function, or physiological behaviour. Does the complex priming of dendritic release^25^, ^26^ interact with phasic spiking, or is the phasic patterning completely lost in the transmission between soma and dendrite, and the diffuse long lasting dendritic signals? At the edges where experiments are challenging, hopefully an accurate model will help.

## AUTHOR CONTRIBUTIONS

**Duncan J. MacGregor:** Conceptualisation; Data curation; Formal analysis; Funding acquisition; Investigation; Methodology; Project administration; Resources; Software; Supervision; Validation; Visualisation; Writing – original draft; Writing – review & editing.

### PEER REVIEW

The peer review history for this article is available at https://publons.com/publon/10.1111/jne.13042.

## Supporting information

Supporting information S1

## Data Availability

The data that support the findings of this study are available from the corresponding author upon reasonable request.
